# Digital learning in medical education: comparing experiences of Malaysian and Japanese students

**DOI:** 10.1186/s12909-021-02855-w

**Published:** 2021-08-04

**Authors:** L. Jun Xin, A. A. Ahmad Hathim, N. Jing Yi, A. Reiko, I. Noor Akmal Shareela

**Affiliations:** 1 Department of Biochemistry, Faculty of Medicine, Universiti Kebangsaan Malaysia (UKM), Jalan Yaacob Latif, 56000 Kuala Lumpur, Malaysia; 2 Department of Culture and Medicine, Shiga University of Medical Science, Seta Tsukinowa-cho, Otsu, Shiga, Japan

**Keywords:** Education, Motivation, Medical, Malaysia, Japan

## Abstract

**Background:**

Medical education has undergone a transformation from conventional to digital learning, enabling learning without any time and place restrictions. Nevertheless, the actual trends of usage and its impact on learning motivation among medical students between developed and developing nations are yet to be investigated. Hence, this study compares the effect of digital learning on learning motivation among Malaysian and Japanese medical students in Universiti Kebangsaan Malaysia (UKM) and Shiga University of Medical Science (SUMS) respectively.

**Methods:**

A modified Students Motivation towards Science Learning (SMTSL) was used to assess the digital learning usage and learning motivation among 150 UKM and 147 SUMS medical students throughout Year 1 to 5.

**Results:**

The frequency of digital learning usage and learning motivation among UKM medical students was significantly higher as compared to SUMS (*p* < 0.001). Electronic books (e-books) were the most preferred source of digital learning among UKM medical students as compared to SUMS medical students who used research articles, e-books, online courses and videos at similar frequencies. UKM medical students in the clinical phase exhibited a significantly higher learning motivation as compared to preclinical students (*p* < 0.05) but not among SUMS medical students.

**Conclusion:**

A suitable learning environment should be developed to encourage digital learning usage among different levels of medical students to enhance its complementary role in medical education and augment the level of motivation among medical students in continuous lifelong learning.

**Supplementary Information:**

The online version contains supplementary material available at 10.1186/s12909-021-02855-w.

## Background

Digital learning is a process of integrating technology-mediated synchronous and asynchronous approaches including assessments, assignments, and tutoring [1], and it enables learning without any time and location restrictions [2]. Digital learning can be divided into a few components, mainly digital teaching materials, digital tools, digital delivery, and autonomous learning [3]. Medical education transformation has successfully incorporated digital learning into its curriculum with virtual courses, simulation software and teleconferencing. Hence, the current medical students are expected to be in the latest trend, by not only learning through traditional methods but to utilise the latest technologies to ensure flexibility in the future dynamic workplace [4].

Looking into undergraduate medical education in Asian countries, a transition from a didactic way of learning to self-directed learning has been inspired by the Western theories [5]. Japan as a developed nation has been promoting the development of e-learning in higher education institutions as part of the e-Japan Initiative under the Japanese Ministry of Education, Culture, Sports, Science, and Technology (MEXT) since 2001. The subsequent ‘IT New Reform Strategy’ in 2006 was aimed to complete national reform through Information Technology (IT) by 2010 to make Japan the front-runner in leading the IT revolution. Following suit, Malaysia, as a developing country started relatively late as a ‘blended learning’ environment, has only been established with the integration of the Learning Management System (LMS) since the launching of the ministry’s initiative, MyHE4.0 (Education 4.0) via the Higher Education Blueprint 2015–2025 [6]. LMS has been utilised in Malaysian higher education institutions mainly for communication purposes, followed by course delivery, productivity, content development, and administration [7]. Looking into statistics, Japan was ranked 18th in the world ‘E-learning Readiness Scoring 2008’ while Malaysia was only in 34th place. Nevertheless, it has been years since the implementation of the above-mentioned policies and the conduction of surveys. Inconclusive findings suggested digital media is the predominant information source for undergraduate medical students [8–10], while others showed that non-digital resources, notably textbook, is the predominant medium of choice for personal study [11]. To date, there is a lack of literature looking into the current actual digital learning usage specifically among medical students in both Malaysia and Japan, leading us to the main aim of this study.

Over the years, the pivot of research on digital learning outcomes in medical education has been revolving around the level of confidence [12–14] and academic achievements or performance [10, 15–18]. A positive correlation between digital learning and learning motivation was found in previous studies but these studies were conducted among undergraduate students studying in courses other than medicine [19–21]. Until recently, there is a lack of studies investigating the correlation between digital learning and learning motivation specifically among medical students. This is indeed crucial as learning motivation can lead to a better outcome of learning [22–24], resilience [24], and thus motivation for lifelong learning [23–25].

Therefore, it is essential to explore and compare the impact of digital learning on learning motivation among medical students between developed and developing nations. This study aims to observe the digital learning culture, by identifying the preferred sources of digital learning, the difference in frequency of digital learning usage and learning motivation, and how digital learning is affecting learning motivation among medical students of the National University of Malaysia, Malaysia (Universiti Kebangsaan Malaysia, UKM) and the Shiga University of Medical Science, Japan (SUMS).

## Methodology

### Study design, study setting, and study population

This was a cross-sectional study involving undergraduate medical students of UKM and SUMS. All UKM and SUMS students undertaking the course of Doctor of Medicine during the period of this study were eligible to participate. All participation from this study is voluntary and respondents have the right to withdraw from this study. Written informed consent is obtained through a declaration of study as part of the questionnaire. Students who did not comprehend English and did not consent to be in this study were all excluded. All data is confidential and only limited to the researchers involved in this study.

### Sampling process

Stratified convenience sampling was used in which the samples were divided by year of study where each year of study contributed to an equivalent ratio to population. The target sample size was 286, determined by identifying the smallest acceptable demographic subgroup in which in this situation our UKM and SUMS medical faculty population size is 1000 with a ± 5% margin of error and a confidence level of 95%. This sample size was also supported by a similar study conducted in Universiti Putra Malaysia, Malaysia with a power of study of 1.000 [26]. Altogether, there were a total of 300 respondents, consisting of 150 and 150 Year 1 to Year 5 UKM and SUMS undergraduate medical students, respectively. However, 3 SUMS respondents were being excluded from this study due to incomplete forms. Thus, the eventual total respondents were 297 with 150 UKM and 147 SUMS respondents, respectively.

### Research instrument

A Students Motivation Towards Science Learning (SMTSL) questionnaire that was developed and validated by a group of researchers from the National Changhua University of Education, Taiwan (Cronbach’s Alpha; α=0.89) was adopted in this study [27]. It consisted of six domains of five-point Likert-scale questions; 7 questions on self-efficacy, 8 questions on active learning strategies, 5 questions on medical learning value, 4 questions on the performance goal, 5 questions on achievement goal, and 6 questions on learning environment stimulation with a total of 35 questions to assess the respondents’ learning motivation. Nine questions were reverse items (Questions 2, 4, 5, 6, 7, 21, 22, 23, 24). Slight modifications with minor grammatical adjustments were made to suit medical students and to avoid confusion. A translated Japanese version of the questionnaire was made by the natives to accommodate SUMS respondents (Cronbach’s Alpha; α=0.87). Based on the total motivation score, respondents were grouped into 3 levels of motivation: “Low”, “Moderate” and “High” by converting into quartile ranks [28]. Respondents in the bottom quartile (25%) were placed in the “Low Motivation” group, the middle 50% were assigned to the “Moderate Motivation” group, and those in the top 25% comprised the “High Motivation” group. Age, year of study, and phase of study among the respondents were also documented as a part of the educational background and demographic data. For the section of preferred digital learning sources and frequency of digital learning usage other than regular class purposes, students were divided into “high usage” (use at least 3 times and above per week), “low usage” (use less than 3 times per week), and “do not use”. A pilot study was done on 35 UKM medical students (Cronbach’s Alpha; α=0.89). Cronbach’s Alpha analysis for SMTSL questionnaire’s subscales for all 35 questionnaire items was shown in Table 1.

**Table 1:** Cronbach’s Alpha Analysis for SMTSL Questionnaire’s Subscales

Variable	Number of Items	Cronbach’s Alpha
Self-Efficacy	7	0.71
Active Learning Strategies	8	0.82
Medical Learning Value	5	0.87
Performance Goal	4	0.84
Achievement Goal	5	0.84
Learning Environment Stimulation	6	0.81
Total	35	0.89

### Procedure and data analysis

A set of questionnaires including an information sheet and consent form were distributed via Google Forms through the social media platform, WhatsApp™ to UKM medical students. As for SUMS medical students, questionnaires were distributed manually by hard copies as formally requested by the Japanese counterparts due to the normalcy of answering written form questionnaires. The study was conducted from August 2019 till February 2020. There was no time limit for survey completion and the scores for each of the scales were calculated. Results were recorded using Statistical Package for Social Science (SPSS) Version 22 and the statistical significance level was set at *p* < 0.05(*), *p* < 0.01(**), and *p* < 0.001(***). Descriptive analyses were included for frequencies of digital learning usage and preferred sources of digital learning in both UKM and SUMS while one-way ANOVA, Student’s t-test, and Chi-Square with post-hoc analysis were used to determine the difference between groups for selected variables.

## Results

### Demographic characteristics

A total of 297, year 1 to 5 undergraduate medical students from UKM and SUMS have participated in this study. 150 (50.5%) students were from UKM and 147 (49.5%) were from SUMS. Based on the total number of students in UKM and SUMS, there was a significant difference (*p* < 0.001) in mean age between UKM (21.67 ± 1.62) and SUMS (24.65 ± 5.65). The distribution of students by year of study is represented in Table 2.

**Table 2:** Student demographic distribution

Year of Study	UKM Number of Students (%)	SUMS Number of Students (%)
1	30 (20)	25 (17)
2	26 (17)	33 (22)
3	27 (18)	32 (22)
4	36 (24)	29 (20)
5	31 (21)	28 (19)
**Total**	150 (50.5)	147 (49.5)

### Analysis of digital learning usage and learning motivation

**Fig. 1:**
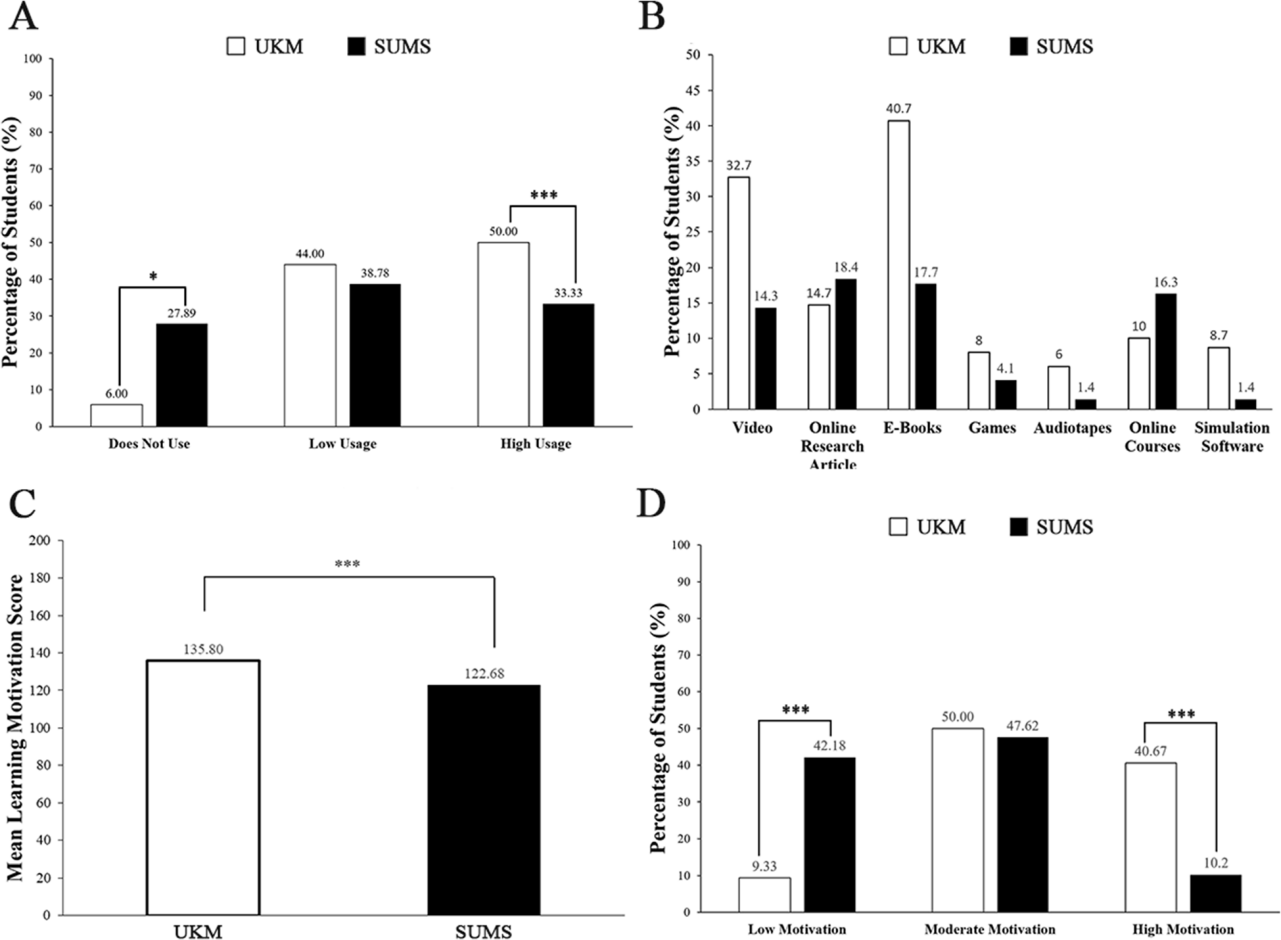
Digital learning usage and learning motivation between Universiti Kebangsaan Malaysia (UKM) and Shiga University of Medical Science (SUMS). (**A**) Digital learning usage between universities, (**B**) preferred learning sources, (**C**) learning motivation score between universities, (**D**) learning motivation group between universities

The frequency of digital learning usage was divided into 3 groups; ‘does not use’, ‘low usage’, and ‘high usage’. In Fig. 1a, most of the respondents from UKM had high digital learning usage (50%) while only 6% did not use digital learning. While in SUMS, most of the respondents had low digital learning usage (38.78%) and 27.89% did not use digital learning. The differences in digital learning usage between UKM and SUMS were analysed with Chi-square analysis and UKM had significantly higher digital learning usage as compared to SUMS (*p* < 0.001). Among those that used digital learning platforms (Fig. 1a, Low Usage and High Usage), UKM students mainly preferred e-books (40.7%) while SUMS medical students who used online resources used research articles (18.4%), e-books (17.7%), online courses (16.3%) and videos (14.3%) at similar frequencies. (Fig. 1b).

UKM students had a significantly higher mean of learning motivation score (*p* < 0.001) as compared to SUMS (Fig. 1c). The score was grouped into ‘Low Motivation’, ‘Moderate Motivation’, and ‘High Motivation’. As shown in Fig. 1d, UKM had a significantly higher percentage (40.7%) of students with high motivation than SUMS (10.2%). Both UKM and SUMS students had moderate motivation at 50.0 and 47.6% respectively. A higher percentage of SUMS students (42.2%) presented with low motivation as compared to UKM (9.3%).

**Fig. 2:**
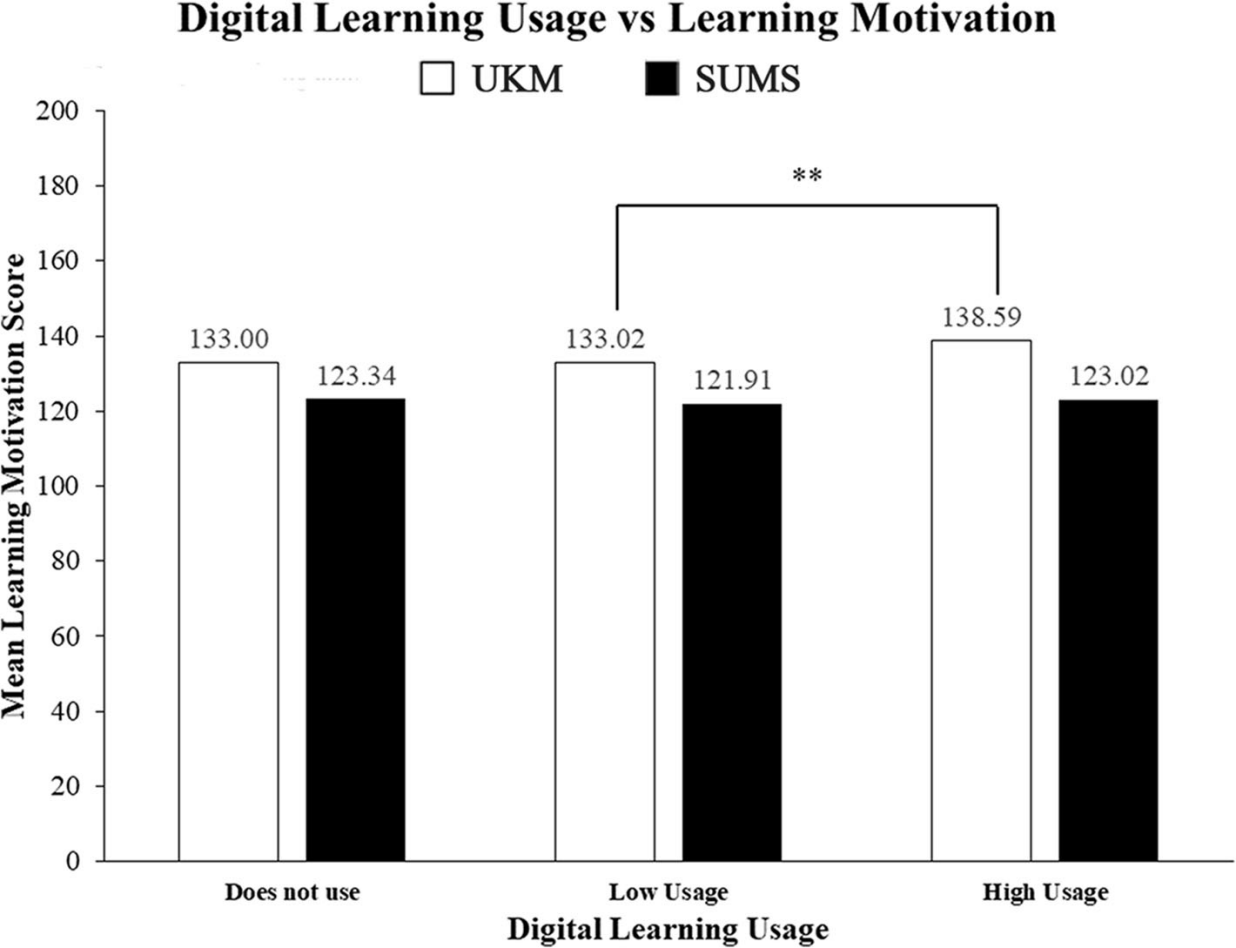
Digital learning usage versus learning motivation between Universiti Kebangsaan Malaysia (UKM) and Shiga University of Medical Science (SUMS)

A direct comparison between learning motivation and digital learning usage was also included in the study. The one-way ANOVA analysis showed that UKM students with high digital learning usage had a significantly higher learning motivation score when compared to low digital learning usage (*p* < 0.05) (Fig. 2). However, all other comparisons between learning motivation score and digital learning usage within UKM and SUMS were not significant.

#### Analysis of learning motivation domains

**Fig. 3:**
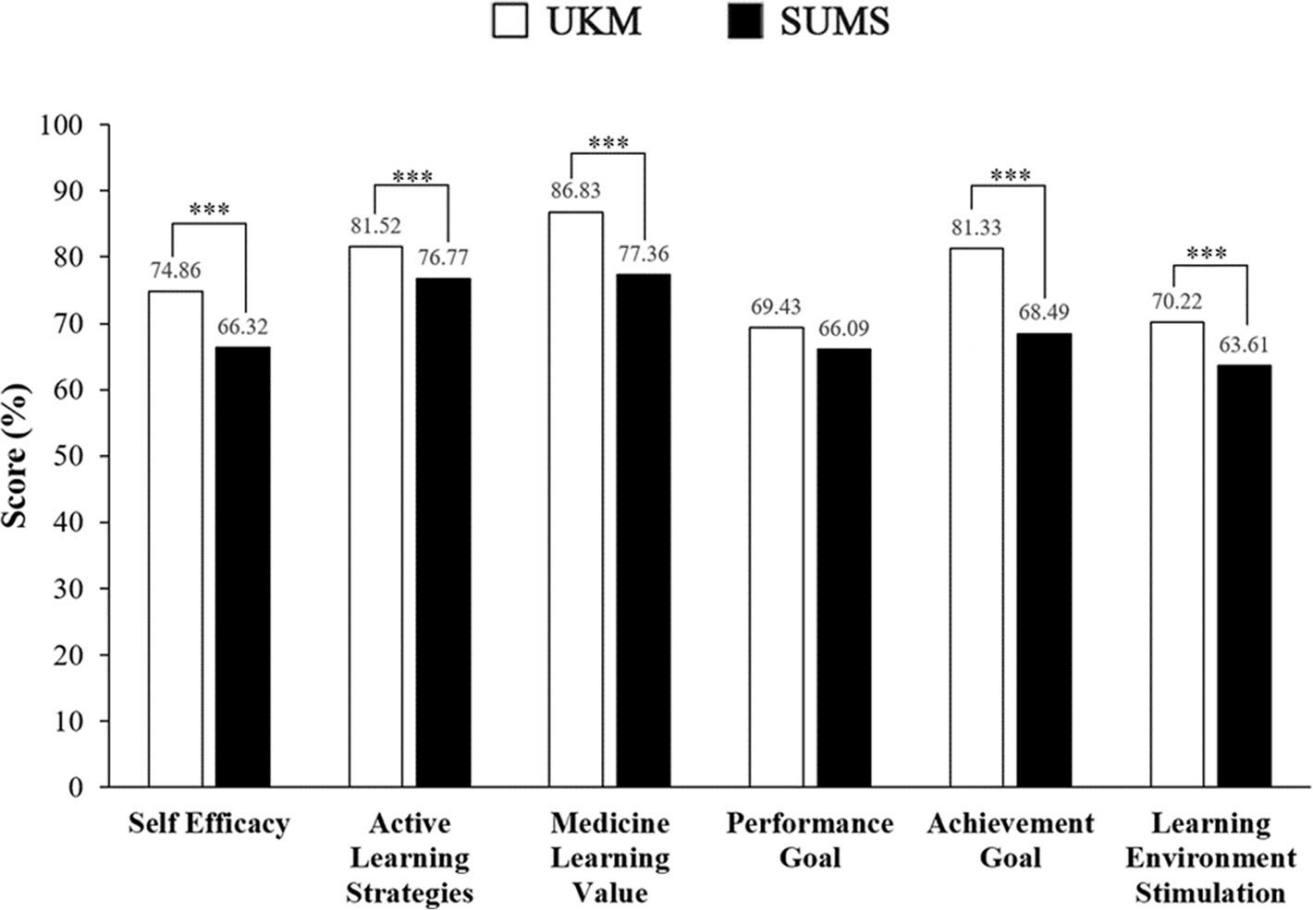
Comparison of learning motivation domains between universities

**Fig. 4:**
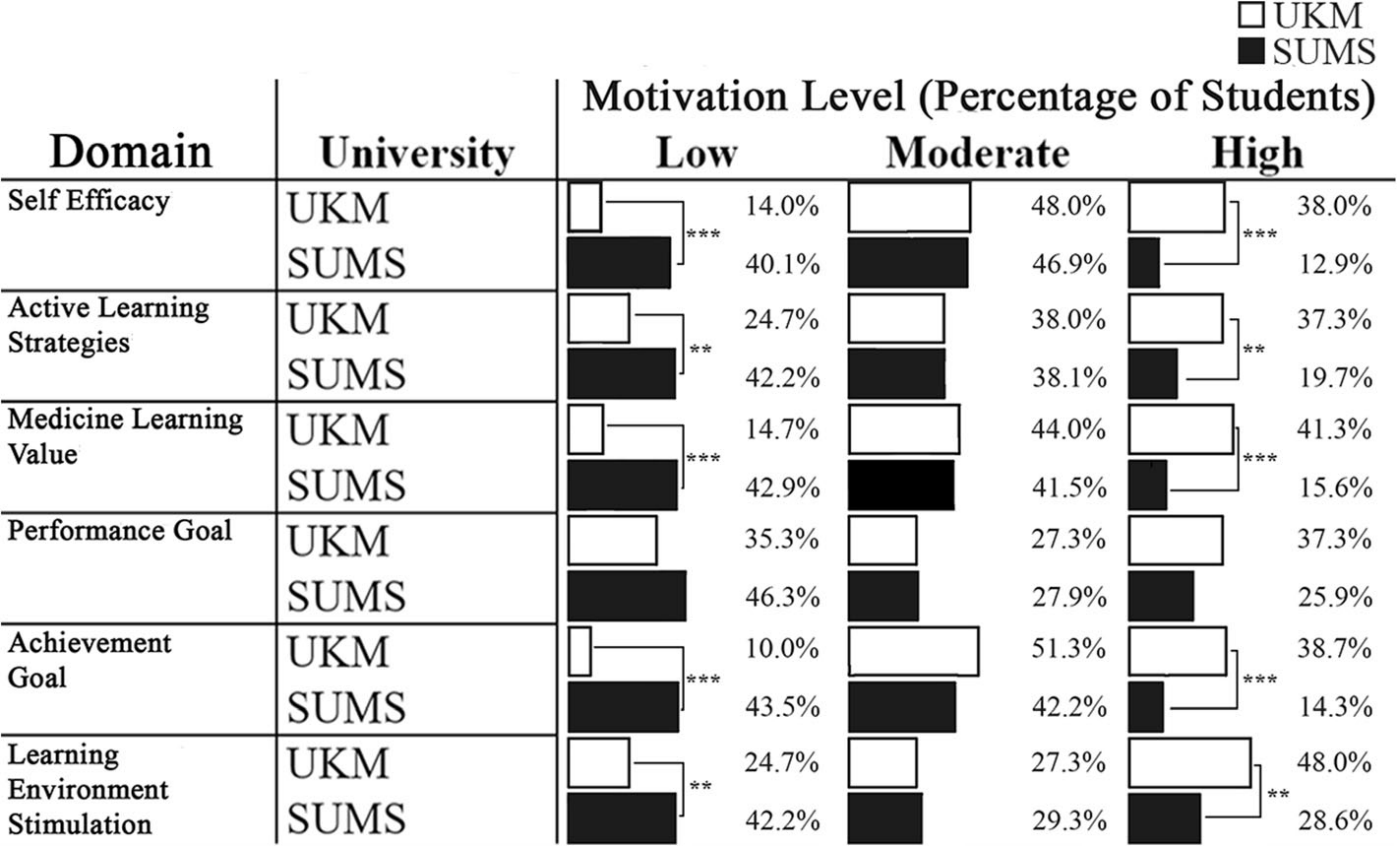
Comparison between Universiti Kebangsaan Malaysia (UKM) and Shiga University of Medical Science (SUMS) medical students based on learning motivation domains and motivation levels

The learning motivation score was divided into 6 domains: self-efficacy, active learning strategies, medicine learning values, performance goal, achievement goal, learning environment stimulation. Mean differences in these domains were studied to see the discrepancies between universities. UKM showed a significantly higher mean score compared to SUMS in all domains except performance goal (Fig. 3). Chi-squared analysis was performed for each domain between universities followed up with residual analysis to find a significant difference between groups. UKM students presented with a significantly higher percentage in both low and high motivation categories for all five domains except for the performance goal when compared with SUMS students (Fig. 4).

### Analysis of digital learning usage and learning motivation among preclinical and clinical students

**Fig. 5:**
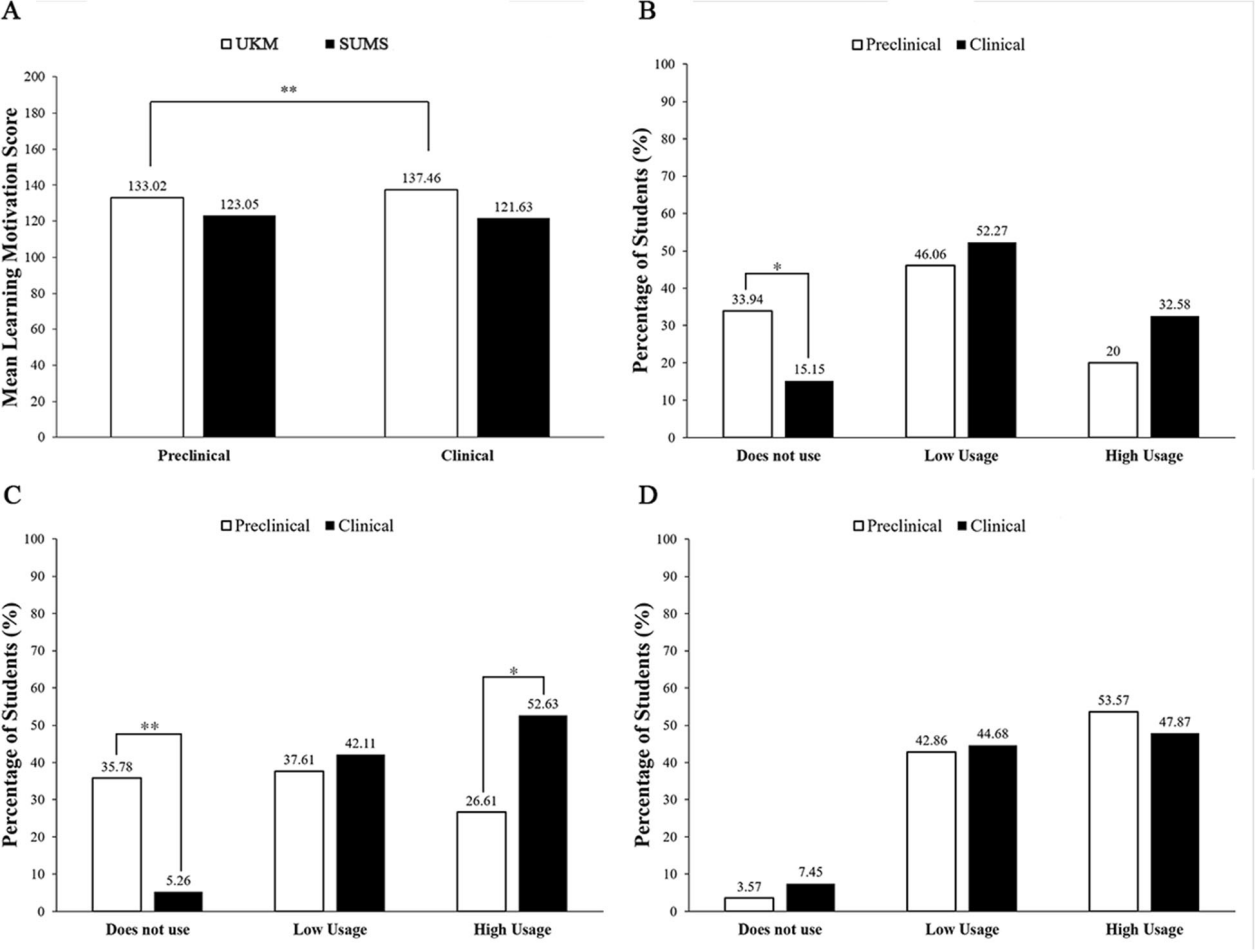
Phase of study with digital learning usage and learning motivation. (**A**) The phase of study against learning motivation score between UKM and SUMS, (**B**) the phase of study against digital learning usage in both UKM and SUMS, (**C**) SUMS phase of study against digital learning usage. (**D**) UKM phase of study against digital learning usage

The phase of study between preclinical and clinical students could influence digital learning usage and learning motivation. Our study found that clinical students in UKM had a significantly higher mean learning motivation score as compared to preclinical students (Fig. 5a). However, there was no significant difference in learning motivation between SUMS preclinical and clinical students. We combined the frequency of using a digital platform from UKM and SUMS from both preclinical and clinical phases, and we found that preclinical students have a significantly higher rate than clinical students in not using a digital platform to supplement their learning (Fig. 5b). When we further analysed the data to each institution, SUMS clinical students were seen to utilise digital platforms more than preclinical students and the rate of preclinical students who did not use digital platforms was significantly higher than clinical students (Fig. 5c). Nevertheless, there was no significant difference in digital learning usage among UKM preclinical and clinical students in all frequencies (Fig. 5d).

**Fig. 6:**
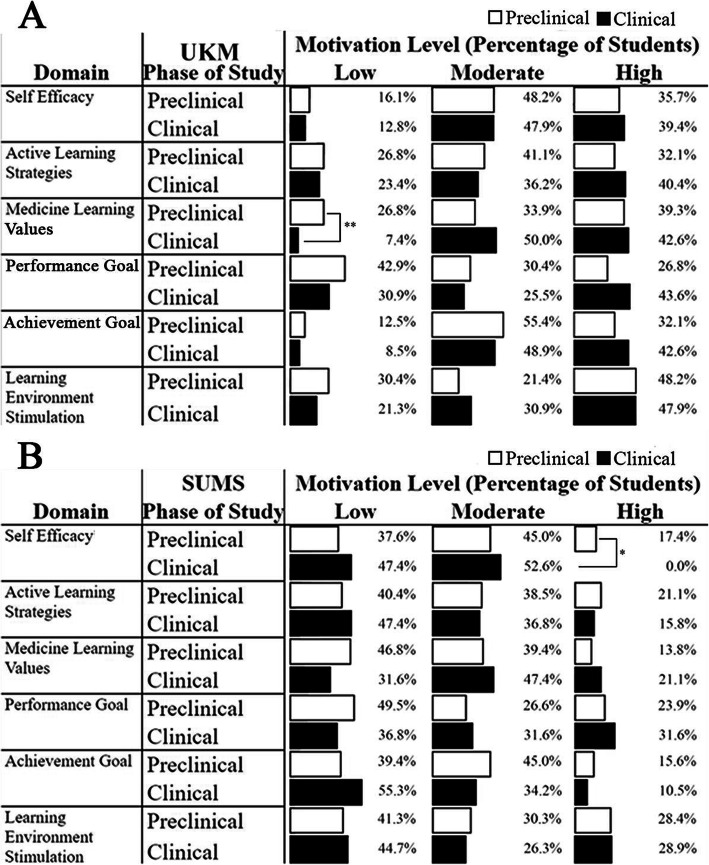
Phase of study learning motivation domains in (**A**) UKM, (**B**) SUMS

We are also interested to seek the comparison of learning motivation domains between phases of the study. There are six domains involved including self-efficacy, active learning strategies, medicine learning values, performance goal, achievement goal, and learning environment simulation. We also divided the students into three levels of motivation: low, moderate, and high. We analysed the data using the Chi-Square test and further tested with residual analysis to identify any significant differences between groups. Among all learning motivation domains, UKM clinical students had a significantly higher medicine learning value (*p* < 0.01) as compared to preclinical students in low motivation level (Fig. 6a). Interestingly, for SUMS, in the high self-efficacy domain, preclinical students exhibited a significantly higher percentage (*p* < 0.05) as compared to clinical students (Fig. 6b).

## Discussion

Information and Communication Technology (ICT) in education aims to support, enhance, and optimize the delivery of knowledge to improve teaching and learning process. It utilizes the Internet, wireless network, mobile phones, computers, software for better means of intercommunication and intra-communication between lecturers and students. Over the years, with the speedy revolution of the Internet, ICT has been widely accessible all over the globe and numerous research on mobile learning are conducted to enhance and upgrade this novel form of learning [19]. Previous statistics in ‘E-learning Readiness Scoring 2008’ suggested that Japan is the front-runner in digital learning as compared to Malaysia [29]. Nevertheless, after more than a decade of the aforementioned survey, our study has found that UKM medical students had significantly higher digital learning usage than SUMS. This is surprising as Japan has all the technological advancements to develop digital learning. The challenge lies in a big gap between government vision and the actual implementation of ICT in medical education [29]. Up until now, classes are still lecture-based, and even with the implementation of the government policies, technologies are being designed in such a way to reinforce the existing conventional facilitated learning, not to transform teaching and learning into individualised self-directed learning [29]. Our findings also revealed that both UKM and SUMS medical students under-utilised simulation software in their studies as evidenced by simulation software being among the least preferred digital learning platforms. In fact, simulation-based education is still novel in the field of medical education and yet to be fully practised to supplement clinical learning [30, 31].

The lack of skills concerning the use of ICT in education among faculty members, lack of maintenance funds, and increasing workloads are among the most cited challenges in developing digital learning in Japan [4, 29, 32]. 81% of medical schools in Japan are actually well-equipped with skills laboratories but the limited time allocation for simulation-based education together with the low motivation among instructors due to inadequate lecturers causing an overload of responsibilities have contributed to the under-development of digital learning in medical education in Japan [30]. Looking into Malaysia’s setting specifically in UKM, advancements have been progressing steadily with the incorporation of digital learning into the medical curriculum via the development of the learning management system, clinical skills lab, and simulation software that encourage student-centred learning [31]. Nonetheless, UKM medical students had the highest preference towards ebooks to supplement their learning as compared to other digital learning platforms. The ease of using ebooks so students can just download them into their mobile devices and access without any time and place restrictions without any need for a physical visit to the library [33]. On the contrary, SUMS medical students use online resources at near similar frequencies. . This could be due to limited resources available as Japanese students prefer textbooks and research articles in the Japanese language. This can be a suggestion to improve SUMS medical curriculum as well as recommending Japanese students to read more international articles to get the latest updates on patient management.

Our findings revealed that learning motivation among UKM medical students was significantly higher than SUMS medical students. An in-depth review of the learning motivation subscale from the SMTSL questionnaire showed that UKM medical students have a significantly higher score in all domains except for the performance goal that showed no significant difference between the two institutions. This signifies that UKM medical students had higher self-efficacy, active learning strategies, medicine learning value, achievement goals, and learning environment stimulation. The lower learning motivation among SUMS medical students could be attributed to the cultural difference causing various personalities as most Japanese students are passive learners and bound to a didactic way of learning [29]. The culture of working part-time among Japanese students [34] as compared to Malaysian medical students who are all full-time students could be one of the factors. Previous studies on the impact of working while studying had shown contradictory results, as students can earn their income and they would become more systematic with increasing working experience [35], but students could become more exhausted, losing their main focus in studies and eventually affecting their academic performance [36]. Learning motivation has been directing behaviour towards achievement and hence, a vital determinant of academic success [37–39]. Since medicine is a program that involves long hours of study, we postulated that the lower learning motivation among SUMS medical students could be contributed by the difficulty in juggling between studies and working, thus affecting their study performance and indirectly their learning motivation. Nevertheless, the insignificant difference in the performance goals between UKM and SUMS medical students can be explained by most medical students having the grit or perseverance of effort in pursuing a medical course as the common ultimate goal is to become a doctor and serve humankind irrespective of their achievement [40].

Our findings on how digital learning can influence learning motivation among UKM medical students supported the results reported by a previous study that demonstrated the same significant positive effect of digital learning on both intrinsic and extrinsic motivation as compared to traditional teaching [11, 28]. This could be explained by five important extrinsic motivation factors in online learning methods, including the learning-teaching process, roles of instructors, participation, and attention, online course environment/technical infrastructure, and time management [28]. Also, it is notable that autonomous learning is closely associated with this learning platform as students who use digital learning often have a high intrinsic motivation [19]. At this stage of understanding, we believe that both students’ intrinsic learning motivation and e-learning’s extrinsic stimulation can contribute to the high learning motivation among constant digital learning users [19]. On the contrary, digital learning usage among SUMS medical students does not show any remarkable association on learning motivation. This can be justified by the slow progression in transforming conventional teaching due to the aforementioned obstacles and the mainstay of government policy that merely emphasizes conventional facilitated learning in the educational system.

Upon an in-depth review of the digital learning usage based on two phases of the study, SUMS preclinical students had significantly higher digital usage as compared to clinical students. This could be due to the curriculum structure of the medical program in SUMS that comprises mainly theoretical studies for the preclinical phase as compared to hospital clerkships with direct interaction with patients in the clinical phase. The almost equal high frequency of using ebooks, online video, online courses and online research articles among SUMS medical students are mainly due to online accessibility. For clinical students, theoretical knowledge is used mainly to apply in the clinical phase of the study. Clinical reasoning with effective communication skills makes up the core of the clinical phase and can only be improved with continuous practice with real patients through hospital clerkship. Until recently, there is still a lack of data that communication with patients could be entirely replaced by digital learning. Hence, digital learning only plays a small part in the delivery of clinical teaching.

For the phase of the study, UKM clinical students showed a significantly higher score in the domain of medicine learning value as compared to preclinical students. As clinical students are dealing with real patients through hospital clerkships more frequently, they could appreciate the learning outcomes in medicine better. For SUMS medical students, preclinical students showed a significantly higher score in the domain of self-efficacy as the preclinical phase in SUMS consists of a longer period of 4 years as compared to 2 years of the clinical phase. Hence, preclinical students have a longer time to adapt and master the learning techniques, making them more confident and boosting their level of self-efficacy. However, to our surprise, none of the SUMS clinical students exhibited high self-efficacy where they are supposedly expected to be confidently in control of their motivation, behaviour, and social environment towards learning. This invites an improvement towards an active learning environment during clinical teaching to promote student confidence.

Our data suggests the trend that high digital learning platform usage independently synchronizes with high learning motivation among both UKM and SUMS medical students. The research outcome can be utilised for the transformation of digital learning in the future especially after the COVID-19 pandemic to replace conventional teaching and learning in medicine as part of a continuous improvement program. Nonetheless, this study encountered certain limitations such as selection bias due to the exclusion of respondents that do not understand English as there may be limited representation in the data towards the Japanese sample population. The lack of other medical faculties participating in this study in both countries may under-represent developing and developed countries. A cultural difference between the two countries may also contribute to the discrepancies in the rating of the responses in the questionnaire.

## Conclusion

Our study result shows a significantly higher digital learning usage in UKM as compared to SUMS, and this provides a good starting point for further studies to explain the relationship between digital learning and learning performance among medical students with regards to the lack of relevant literature and discussion. Since our data suggested high digital learning usage is in trend with high learning motivation in this modern era of technology, continuous improvement in medical curriculum especially in establishing new approaches towards online active learning has to be explored, especially during the current COVID-19 pandemic [41–44] which imposes restrictions to conventional teaching and learning methods to nurture medical graduates who are tech-savvy and life-long learners.

## Supplementary Information

**Additional file 1.**

## Data Availability

All data and materials are available from the corresponding author by request.
